# Comorbidities and Quality of Life in Women Undergoing First Surgery for Endometriosis: Differences Between Chinese and Italian Population

**DOI:** 10.1007/s43032-021-00487-5

**Published:** 2021-03-09

**Authors:** Huixi Chen, Silvia Vannuccini, Tommaso Capezzuoli, Marcello Ceccaroni, Liu Mubiao, Huang Shuting, Yanting Wu, Hefeng Huang, Felice Petraglia

**Affiliations:** 1 Obstetrics and Gynecology, Department of Experimental and Clinical Biomedical Sciences, Careggi University Hospital, University of Florence, Largo Brambilla, 3, 50134 Florence, Italy; 2 International Peace Maternity and Child Health Hospital, School of Medicine, Shanghai Jiao Tong University, Shanghai, China; 3 Shanghai Key Laboratory of Embryo Original Disease, Shanghai, China; 4 Department of Molecular and Developmental Medicine, University of Siena, Siena, Italy; 5 Gynecology and Obstetrics, Gynecologic Oncology, Minimally-Invasive Pelvic Surgery, International School of Surgical Anatomy, IRCCS Sacro Cuore Don Calabria Hospital, Negrar di Valpolicella, Verona, Italy; 6 Guangdong Provincial People’s Hospital, Guangdong Academy of Medical Sciences, Guangdong, China

**Keywords:** Autoimmune disorders, Comorbidities, Endometriosis, Ethnicity, Inflammatory diseases, Metabolic disorders, Psychiatric diseases, Quality of life

## Abstract

An observational cross-sectional study was conducted in a group (*n* = 371) of fertile age women with endometriosis, by administering a structured questionnaire, in order to evaluate the incidence of gynecological and systemic comorbidities and the impact on quality of life (QoL) in two different groups of Italian and Chinese patients affected by endometriosis. Chinese (*n* = 175) and Italian (*n* = 196) women were compared regarding systemic (inflammatory, autoimmune, and mental) and gynecological comorbidities, pain symptoms, and QoL, by using the Short Form 12 (SF-12). Italian patients resulted younger at the diagnosis and suffered more frequently from severe pain than Chinese ones. Deep infiltrating endometriosis (DIE) and mixed phenotypes were more frequent in Italian patients, whereas ovarian (OMA) and superficial endometriosis (SUP) were more common in the Chinese. The Italian group showed more systemic comorbidities, and those disorder were already present before the diagnosis of endometriosis. Furthermore, the Italian group showed lower SF-12 physical and mental scores, suggesting a worse health-related QoL in Italian endometriotic patients. A number of differences has been observed between Italian and Chinese women with endometriosis in terms of comorbidities and QoL, which may be related to the ethnicity, the different health system organization and the social and cultural background.

## Introduction

Endometriosis is a benign gynecological disease characterized by an estrogen-dependent inflammatory process with ectopic localization of endometrial cells, affecting approximately 10% of reproductive aged women. Debilitating pain-related symptoms (dysmenorrhea, dyspareunia, dysuria, and dyschezia) and infertility are commonly observed in affected women. A negative association exists between endometriosis-associated pain and daily activities, self-care, and productivity at work up to job loss, and these may cause a low quality of life [1]. Medical or surgical treatment of endometriosis aim to achieve a complete and durable symptom relief, with good physical and mental health [2]; however, the disease is chronic and recurrent [3].

Furthermore, endometriosis is associated with gynecological [4] and systemic comorbidities, including immune (asthma, rheumatoid arthritis, psoriasis, and multiple sclerosis), inflammatory (bowel inflammatory disease and Crohn’s disease), and psychiatric disorders (depression and anxiety ) [5–7].

The majority of epidemiological data on endometriosis comes from reports on Caucasian women [8]. However, also Asian women are likely to be diagnosed with endometriosis, whereas African-American women are less frequently affected [9–11]. Few evidences are available on the Chinese population [12], but there is a high number of patients with endometriosis in China because of the large population base. Moreover, information on women with endometriosis differentiated by ethnicity are quite limited. The present study aims to evaluate the difference between Chinese and Italian population with endometriosis in terms of symptoms, comorbidities, and Quality of Life (QoL).

## Materials and Methods

An observational cross-sectional multicenter study was conducted in a group (*n* = 371) of fertile age women (25–45 years old) with endometriosis, recruited in four different hospitals, all third level centers for endometriosis treatment (Florence and Negrar di Valpolicella, Verona, Italy; Shanghai and Guangdong, China). They were divided into two age-matched groups based on the ethnicity: (a) Chinese population (*n* = 175) and (b) Italian population (*n* = 196). We included only women with histological diagnosis of endometriosis. Data were collected within 2 years after a single surgery for endometriosis through a structured questionnaire, during a clinical follow-up visit. Women with previous or actual pregnancy were excluded. Also women with multiple surgical interventions for endometriosis were not included in the study. A database was built collecting for each case all the following information:

- demographic characteristics (age, body mass index [BMI], and age at menarche);
- endometriosis data: age at the diagnosis of endometriosis, phenotype of endometriosis at surgery (ovarian endometriosis [OMA], deep infiltrating endometriosis [DIE], superficial endometriosis [SUP], and mixed phenotype), if hormonal medical treatment before and after surgery was administered, and current symptoms (dysmenorrhea, dyspareunia, non-menstrual pelvic pain, and urinary pain), measured as absent, mild, moderate, and severe, according to the visual analog scale (VAS). In fact, women graded their perception of each type of pain on a 10-cm line from 0 (no pain) to 10 (unbearable pain); a mean VAS score of 7 or higher was considered severe;
- gynecological (uterine fibroids, adenomyosis, and polycystic ovarian syndrome) and systemic comorbidities, including autoimmune (thyroiditis, rheumatoid arthritis, psoriasis, pemphigus, multiple sclerosis, and myasthenia gravis), metabolic (obesity, hypertension, and hypercholesterolemia), inflammatory (allergic rhinitis, allergic asthma, irritable bowel syndrome, and inflammatory bowel diseases), and mental health diseases (depression, anxiety and panic disorder, eating disorders); it has been also asked whether the disorder appears before or after the diagnosis of endometriosis;
- evaluation of health-related Quality of Life (QoL) by using the Short Form-12 (SF-12) [13].

The data were validated through review of medical records of all participants to confirm what was reported by patients, especially for systemic comorbidities. The study was approved by the locals Institutional Review Boards, and all participants provided written informed consent to be included in the series.

### Statistical Analysis

Statistical analysis was performed using IBM SPSS Statistics software, version 22 (IBM Corporation, Armonk, NY, USA). Statistically significant differences between groups were determined using Student's *t*-test for continuous variables and chi-square test or Fisher's exact test for categorical variables. A *p* value <0.05 was considered statistically significant. The sample size was estimated to detect differences of at least 0.5 standard deviations in quantitative variables or 20% in the frequency of categorical variables between groups with 80% statistical power and 95% confidence level. In order to achieve a statistical power of 95%, we needed at least 111 patients with a significance level of 0.05 and an effect size of 0.3.

## Results

The group of Italian women resulted younger than the Chinese at diagnosis of endometriosis (*p* < 0.0001). Chinese women were more frequently diagnosed with OMA (84.0% vs 46.7%), whereas Italians had significantly more DIE (4.1% vs 1.8%) and mixed phenotypes (40% vs 5.6%) (*p* < 0.0001) (Table 1). Furthermore, the group of Italian women underwent more frequently to medical hormonal treatment before surgery (21% vs 43.3%, *p* < 0.0001). On the contrary, no differences were found in terms of indications for surgery, which was mainly represented by pain symptoms (Table 1).

**Table 1:** Clinical characteristics of the two populations

	Chinese (*n* = 175)	Italian (*n* = 196)	*p* value
Age (years)	36.5±6.7	36.3±6.4	0.769
BMI	21.4±3.1	22.2±3.3	0.021
Menarche (years)	13.5±1.6	12.3±1.3	<0.0001
Age at first diagnosis (years)	32.2±5.9	27.3±5.6	<0.0001
Medical treatment before first surgery	34 (21.0%)	85 (43.4%)	<0.0001
Indication for first surgery			
Pain	92 (54.8%)	104 (53.1%)	0.025
Pain and infertility	7 (4.2%)	24 (12.2%)	
Infertility	8 (4.8%)	4 (2%)	
Imaging	61 (36.3%)	64 (32.7%)	
Endometriosis phenotype at surgery			
OMA	137 (84.0%)	91 (46.7%)	<0.0001
DIE	3 (1.8%)	8 (4.1%)	
SUP	14 (8.6%)	14 (7.2%)	
OMA+DIE	4 (2.5%)	26 (13.3%)	
OMA+DIE+SUP	0 (0.0%)	25 (12.8%)	
OMA+SUP	5 (3.1%)	24 (12.3%)	
DIE+SUP	0 (0.0%)	7 (3.6%)	
Dysmenorrhea			
No	52 (36.6%)	60 (31.7%)	<0.0001
Mild	56 (39.4%)	34 (18.0%)	
Moderate	14 (9.9%)	52 (27.5%)	
Severe	20 (14.1%)	43 (22.8%)	
Dyspareunia			
No	94 (76.4%)	57 (29.7%)	<0.0001
Mild	24 (19.5%)	43 (22.4%)	
Moderate	3 (2.4%)	66 (34.4%)	
Severe	2 (1.6%)	26 (13.5%)	
Non menstrual pelvic pain			
No	92 (66.2%)	57 (29.5%)	<0.0001
Mild	35 (25.2%)	50 (25.9%)	
Moderate	10 (7.2%)	56 (29.0%)	
Severe	2 (1.4%)	30 (15.5%)	
Urinary pain			
No	123 (88.5%)	142 (74.0%)	0.011
Mild	11 (7.9%)	32 (16.7%)	
Moderate	5 (3.6%)	16 (8.3%)	
Severe	0 (0.0%)	2 (1.0%)	
Medical treatment after surgery	130 (60.9%)	154 (78.2%)	0.3904

Values are mean + SD or *n* (%)

*BMI* body mass index, *OMA* ovarian endometriosis, *DIE* deep infiltrating endometriosis, *SUP* superficial endometriosis

Regarding gynecological comorbidities, PCOS (7.9% vs 5.3%) and uterine fibroids (9.3% vs 3.2%) were more common in the Chinese than in Italian patients, while adenomyosis was more common in Italians (27.9% vs 9.9%). Most of the systemic comorbidities observed were more common in Italian women than in the Chinese, i.e., autoimmune diseases (27.5% vs 4.6%, *p* < 0.0001), metabolic/endocrine diseases (14.8% vs 8.7%, *p* = 0.007), inflammatory diseases (44.5% vs 19.9%, *p* < 0.0001), and mental health disorders (35.3% vs 3.7%, *p* < 0.0001) (Table 2). Among the comorbid autoimmune diseases, the Italian population showed more thyroiditis (17.9% vs 3.4%), dermatological diseases (psoriasis and pemphigus) (4.1% vs 0) and neuromyopathies (multiple sclerosis and myasthenia gravis) (2.6% vs 0) than the Chinese population. Similarly, some of the comorbid metabolic/endocrine diseases were more common in Italian patients including hypertension (7.7% vs 1.7%) and obesity (3.6% vs 0), as well as some inflammatory diseases, i.e., allergic asthma (1.5% vs 0) and intestinal inflammatory diseases (3.6% vs 0). Also mental health disorders were more common in Italian patients, i.e., depression (19.4% vs 1.1%), anxiety (10.7% vs 1.7%), and anorexia (1.5% vs 0). Whether the comorbidities were identified before or after the diagnosis of endometriosis was also evaluated, and Italian women have already been diagnosed with a systemic disease before endometriosis identification (Table 2).

**Table 2:** Gynecological and systemic comorbidities of endometriosis in the two populations

	Chinese (*n* = 175)	Italian (*n* = 196)	*p* value
Gynecological comorbidities			
No	104 (68.9%)	115 (60.5%)	<0.0001
PCOS	12 (7.9%)	10 (5.3%)	
Adenomyosis	15 (9.9%)	53 (27.9%)	
Uterine fibroids	14 (9.3%)	6 (3.2%)	
Multiple	6 (4.0%)	6 (3.2%)	
Before endometriosis diagnosis	43/65 (91.5%)	3/25(4.0%)	<0.0001
Autoimmune diseases	8 (4.6%)	51 (26.0%)	<0.0001
Before endometriosis diagnosis	1/7 (14.3%)	36/51 (70.6%)	0.007
Metabolic/endocrine diseases	28 (8.7%)	39 (14.8%)	0.007
Before endometriosis diagnosis	2/13 (15.4%)	14/26 (53.8%)	0.023
Inflammatory diseases	46 (19.9%)	95 (44.5%)	<0.0001
Before endometriosis diagnosis	10/16 (62.5%)	68/78 (87.2%)	0.027
Mental health disorders	5 (3.7%)	77 (35.3%)	<0.0001
Before endometriosis diagnosis	1/5 (20.0%)	45/65 (69.2%)	0.044

Values are mean + SD or *n* (%).

*PCOS* polycystic ovary syndrome.

**Fig. 1:**
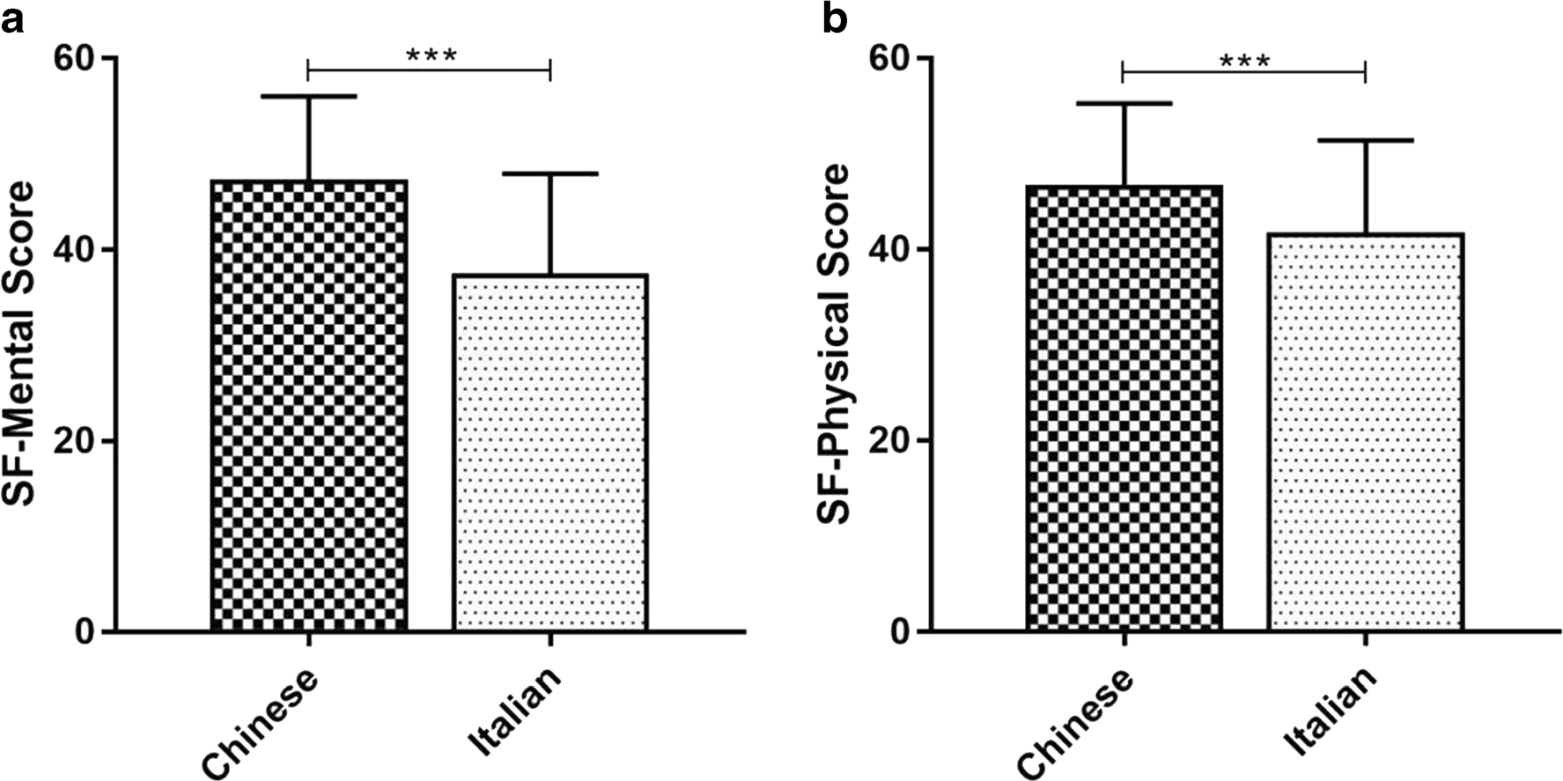
The histograms showed the SF-12 physical and mental scores in Chinese and Italian population, respectively. ***p < 0.001

No differences were found in terms of medical treatment after surgery between the two groups (60.2% vs 78.2%, *p* = 0.3904). Regarding pain symptoms at the follow-up visit, Italian patients suffered more frequently from severe pain than the Chinese, in terms of dysmenorrhea (*p* < 0.0001), dyspareunia (*p* < 0.0001), non-menstrual pelvic pain (*p* < 0.0001), and dysuria (*p* = 0.011) (Table 1). Accordingly, the evaluation of QoL in the two groups showed that the Chinese patients had both for SF-12 physical score (46.5 ± 8.7 vs 41.5 ± 9.9, *p* < 0.0001) and SF-12 mental score (47.1 ± 8.8 vs 37.2 ± 10.7, *p* < 0.0001) higher than the Italian patients (Fig. 1). Stratifying patients according to endometriosis phenotypes, those with DIE among Italians have significantly worse mental scores compared to Chinese (36.8 ± 10.4 vs 49.8 ± 8.2, *p* = 0.010), whereas physical scores were not significantly different (41.9 ± 7.3 vs 44.6 ± 13.1, *p* = 0.649), although the sample size in Chinese population is very small. Regarding women with only ovarian localization of endometriosis among Italians and Chinese, we confirmed that Italians reported more severe pain symptoms (dysmenorrhea 26.7% vs 10.5%; dyspareunia 13.5% vs 1%; non-menstrual pelvic pain 16.5% vs 0%) and a worse QoL (SF-12 physical score 41.3 ± 11.8 vs 46.7 ± 8.5, *p* = 0.010; SF-12 mental score 38.5 ± 10.9 vs 47.4 ± 9.0).

## Discussion

The present cross-sectional and multicentric study explored for the first time the difference in endometriosis phenotypes, comorbidities, and QoL between Italian and Chinese patients with history of a single surgery for endometriosis. The Italian endometriotic patients showed more frequently DIE and mixed phenotypes and systemic and gynecological comorbidities than the Chinese patients. In addition, the Italian group presented at the follow up visit within 2 years after surgery with lower SF-12 physical and mental scores and more severe pain symptoms after stratifying for endometriosis phenotype and despite no differences in terms of post-surgery hormonal medical treatment.

Generally, endometriosis has been considered as a disease of Caucasian and middle-class women who delayed children-bearing and, at the beginning, the majority of the studies were performed in Caucasian population. In the last years, a number of studies have been published on the Chinese population and differences in genetic mutations that predispose Asian and Western populations to endometriosis have been found [14]. Furthermore, genome wide association studies reported that among North Chineses polymorphisms in rs12700667 located within the intergenic region of 7p15 are associated especially with an elevated risk of OMA, which is consistent with the higher incidence of OMA found in our study [15]. On the contrary, the Italian group showed more frequently DIE and mixed phenotypes, which is consistent with worse pain symptoms and lower QoL scores [16, 17]. We may hypothesize that this finding may be due to a more accurate pre-operative imaging diagnosis, which contributes to the laparoscopic identification of deep lesions and to the adequate site-mapping of endometriosis [18]. A number of Italian gynecologists devoted to endometriosis management are themselves experts in imaging for endometriosis, which improves the pre-operative diagnosis, especially of DIE lesions [19]. This difference may account for an underestimation of DIE in Chinese. However, the higher incidence of OMA among Chinese is in line with previous reports [15], and it is also consistent with their better QoL, given the lower association of this phenotype with pain symptoms, if DIE is not associated [20]. Conversely, the DIE phenotype is more frequently linked to a more severe and aggressive clinical presentation, due to specific pathogenic mechanisms such as a highly decreased apoptosis, an increased proliferation activity related to oxidative stress, a higher expression of metalloproteinases and activins for invasiveness and a relevant activation of neuroangiogenesis in ectopic endometrial lesions, compared to other phenotypes [21].

Furthermore, the two groups have significantly different baseline characteristics, such as younger age at diagnosis and more comorbidities in Italian women. No differences were found in terms of indications for surgery, with a similar rate of pain symptoms or imaging findings (i.e., adnexal mass) among the two groups. In addition, the study involved only referral hospitals for endometriosis with expertise in both surgical and medical treatment of the disease. Their protocols for endometriosis management were similar, following international guidelines [14, 22, 23]: depending on age, pregnancy desire, pain symptoms, endometriosis phenotype, and each woman’s wishes, an individualized management was planned. Younger age at surgery may denote, on one hand, a more aggressive presentation of the disease, whereas on the other it may contribute to more severe symptoms and worse QoL in Italian women with endometriosis [2]. A more relevant impact of endometriosis in Italian than Chinese women may be also explained by the difference in the medical approach, in the access to health care, in the acceptance to medical treatment between the two populations. Our study showed that Chinese underwent less frequently to a pre-operative medical hormonal treatment, whereas the rate of post-surgical hormonal treatment was approximately the same. Given the low pain scores and the better quality of life in Chinese compared to Italian group in our study, a potential role played by the Traditional Chinese Medicine (TCM) should be considered. TCM is commonly used in China to provide pain relief control, the recurrence of endometriosis following surgery, and to improve QoL [24–27]. TCM users were also less likely to require surgical treatment for endometriosis than non-users [25] and among those operated was effective in controlling recurrences [26, 27]. However, although TCM is often used for the management of endometriosis patients, there is a lack of high quality clinical evidence supporting its effectiveness compared to other medical treatments. However, as data on the use of TCM missing in our data, its potential role in contributing to pain control and to postpone first surgical intervention should be taken into account.

In other multicenter studies it was shown that ethnicity influenced the access to health care, diagnosis and treatment for endometriosis [9], thus, as a exposure factor, ethnicity throws its effect on clinical presentation and management through social and cultural constructs rather than genetics [28]. Various components of pelvic pain are the primary symptoms of endometriosis and several studies support that individual conceptualization of pain may be affected by different social cultures in different races [28–30]. Asian patients tend to normalize pain, while Caucasian patients more likely to seek health care positively [31]. A cross-country study reported that the incidence and the intensity of pain symptoms were significantly lower in Chinese population than Russia and France [32]. In our study, Italian patients with endometriosis suffered more from severe pelvic pain than Chinese women, suggesting a different cultural background rather than actual pain conceptualization. Also the different health system organization, with less coverage for outpatient clinic and primary care in China determining a less request for medical care, entails a diverse approach to such a chronic and multifaceted disease.

Furthermore, Italian women presented more commonly with systemic comorbidities, especially inflammatory diseases and mental health disorders (such as depression and anxiety). On the contrary, in the Chinese population, the most common comorbidities were gynecological (PCOS and uterine fibroids). Despite the prevalence of comorbidities resulted significantly different between the two groups, the diagnostic criteria of each disorders were well defined according to the current and updated guidelines, which stands for all over the world. Furthermore, the systemic comorbidity reported by the patient during medical history collection was always confirmed by reviewing medical records. Regarding the potential differences between Italy and China overall in terms of incidence of comorbidities, according to the current relevant literature there are no significant differences between the two population in terms of baseline incidence of autoimmune, inflammatory, and metabolic diseases [33, 34]. Besides, data on comorbidities among Italians are consistent with figures reported in already published studies on endometriosis [5, 35–38]. The different distribution of comorbidities with endometriosis in the two population suggested a different spectrum of diseases under different society and culture. A better identification of systemic comorbidities should be taken into account in Italian group, because of the presence of a multidisciplinary team—including an immunologist, a gastroenterologist, a psychiatrist, a dietician, a neurologist, a pain specialist and a physical therapist—in the Italian Endometriosis centers. However, figures from Italians are absolutely in line with those reported in studies on cohorts from all over the world on women with endometriosis [5, 35–38]. A better investigation on systemic comorbidities among Chinese may be desirable in order to identify potential diseases accompanying endometriosis, in order to better explain clinical presentation and to plan a fully comprehensive treatment [39]. This would also allow to increase the awareness of endometriosis among specialists of other disciplines. Moreover, these comorbidities were mostly diagnosed before endometriosis, suggesting a complex inner correlation between a number of systemic diseases and predisposition to endometriosis development [5]. However, given the cross-sectional study design and a short time period of observation since the first operation for endometriosis, it is not possible to account for potential comorbidities would have developed later.

SF-12 is a validated instrument for quantifying the Health-Related Quality Of Life (HRQOL) and higher scores on the SF-12 physical component summary (PCS) and mental component summary (MCS) indicate better quality of life. The SF-12 scores of PCS and MCS were significantly higher in Chinese than in Italian patients with endometriosis, suggesting that the QoL in the Chinese patients resulted better than in the Italian patients and this does not depend on the phenotype of endometriosis. In fact, after stratifying for DIE and OMA phenotype, data on pain symptoms intensity and QoL among Chinese women were still better than in the Italian Group. As already mentioned, a potential role of TCM should be considered, even though no data are available on that variable. Probably, the better scores among Chinese patients are in part related to the lower number of comorbidities in the Chinese women, suggesting a possible poor attention to this kind of questioning when clinical history was collected. A study reported that QoL was independently associated with more severe dysmenorrhea and more severe chronic pelvic pain, but not with higher ASRM stage [40, 41]. A correlation between different phenotypes of endometriosis and levels of stress perception was observed [1, 42], suggesting a possible association between the forms of endometriosis and impact on QoL. Besides, the presence of psychiatric diseases was related to endometriosis-associated pain but not with lesions localization [43, 44]. It is clear that the adverse effect of endometriosis on QoL are related to various factors, including pain/discomfort symptoms, infertility, and high intensity of stress perception [1, 45, 46]. In addition, ethnicity may affect quality of life of endometriotic patients through different pain conceptualization and access and acceptance to health care.

Some limitations and strengths of the study should be acknowledged. The study included only women with endometriosis who underwent first surgery in referral centers for endometriosis. Currently, a large number of patients with endometriosis are only medically treated with good results and the first-line approach for endometriosis symptoms should be medical treatment [47]. However, the study is focused only on those with a previous surgical operation for endometriosis, so our results cannot be applied to all endometriosis-affected women. The choice of including only women with histological diagnosis of endometriosis aimed to have strict criteria of inclusion for the centers involved in the research, in order to minimize bias due to different imaging techniques and no universally shared diagnostic criteria for the non-invasive diagnosis of each phenotype of endometriosis. Women with history of multiple surgery were excluded, in order to reduce the impairment of QoL due to repeated surgery, relapses, and recurrences. In addition, as the study is cross-sectional it is not possible to predict which comorbidities would have developed as time passes.

Based on the results, in young women with comorbid diseases and mild symptoms, the possibility of endometriosis should be considered in order to not delay the diagnosis of endometriosis. Moreover, more and more patients with endometriosis-associated pain required a better treatment of the various comorbidities in order to improve their quality of life.
